# Frequency and predictors of disease progression in contemporary ACPA-negative undifferentiated arthritis in a treated-to-target early arthritis clinic

**DOI:** 10.1136/rmdopen-2026-007443

**Published:** 2026-10-27

**Authors:** Ludovico De Stefano, Elena Manna, Clelia Zampaglione, Federica Sabatelli, Emmanuele Guadalupi, Garifallia Sakellariou, Antonio Manzo, Carlomaurizio Montecucco, Serena Bugatti

**Affiliations:** 1 Department of Internal Medicine and Therapeutics, University of Pavia, Pavia, Italy; 2 Division of Rheumatology, IRCCS Policlinico San Matteo Foundation, Pavia, Italy; 3 S.C. Reumatologia, ASST Grande Ospedale Metropolitano Niguarda, Milan, Italy; 4 Istituti Clinici Scientifici Maugeri IRCCS, Pavia, Italy

**Keywords:** Rheumatoid Arthritis, Anti-Citrullinated Protein Antibodies, Rheumatoid Factor, Outcome Assessment, Health Care, Methotrexate

## Abstract

**Objectives:**

Since the introduction of the 2010 American College of Rheumatology/European Alliance of Associations for Rheumatology (EULAR) criteria, undifferentiated arthritis (UA) has become predominantly seronegative and heterogeneous, with poorly defined predictors of persistence. We assessed rheumatoid arthritis (RA) development and treatment intensification in contemporary anticitrullinated protein antibodies (ACPA)-negative UA managed under a standardised treat-to-target protocol.

**Methods:**

Consecutive ACPA-negative patients with UA enrolled in the Pavia Early Arthritis Clinic (2011–2019) were analysed. UA was defined as clinical synovitis not fulfilling 1987 or 2010 RA criteria and without alternative diagnosis at baseline or within 6 months. All patients started hydroxychloroquine; escalation was driven by persistent disease activity. Outcomes over 24 months were RA development and the composite outcome of RA development or treatment intensification.

**Results:**

Among 402 patients, arthritis was predominantly hand-restricted (75.1%), less often combined hand/large-joint (19.2%) or isolated large-joint (5.7%). RA developed in 13.1% of the patients, whereas 38.1% developed RA or required treatment intensification. Combined hand and large-joint arthritis independently predicted RA development (adjusted OR 2.68, 95% CI 1.29 to 5.57) and the composite outcome (adjusted OR 3.32, 95% CI 1.77 to 6.24). C reactive protein (CRP) elevation and pain burden were associated with treatment intensification. Classification tree analysis identified large-joint involvement as the first discriminator; together with the no-large-joint/CRP >0.5 mg/dL branch, it captured 64% of events, with positive predictive value 48% and negative predictive value 72%.

**Conclusions:**

In contemporary ACPA-negative UA, progression is driven by anatomical joint distribution rather than generic disease activity. Combined hand/large-joint involvement identifies a high-risk phenotype, while CRP refines risk in clinically milder branches.

**WHAT IS ALREADY KNOWN ON THIS TOPIC**

- After the 2010 American College of Rheumatology/European Alliance of Associations for Rheumatology (EULAR) criteria, undifferentiated arthritis (UA) is predominantly seronegative and often mild, but a subset progresses or remains persistent and conventional predictors are poorly informative.

**WHAT THIS STUDY ADDS**

- In a strictly anticitrullinated protein antibodies-negative UA cohort managed with a standardised treat-to-target protocol, combined hand/large-joint synovitis predicted rheumatoid arthritis development and treatment intensification better than generic joint counts, while C reactive protein (CRP) refined risk among patients without large-joint disease.

**HOW THIS STUDY MIGHT AFFECT RESEARCH, PRACTICE OR POLICY**

- Simple baseline anatomical patterns and CRP may help stratify follow-up and treatment escalation in UA when autoantibodies are absent and advanced imaging is not routinely available.

The recognition that the development of rheumatoid arthritis (RA) proceeds through sequential stages of risk factors, asymptomatic immune dysregulation, symptoms and subclinical joint inflammation,^1^ although not invariably following the same trajectory in all patients,^2^ has profoundly influenced clinical practice and research over the past decade. Following the introduction of the American College of Rheumatology (ACR)/European Alliance of Associations for Rheumatology (EULAR) 2010 criteria,^3^ virtually all patients positive for anticitrullinated protein antibodies (ACPA) who develop clinical synovitis are classified as RA and recommended for prompt treatment with methotrexate (MTX).^4^ As a result, ACPA-positive undifferentiated arthritis (UA) has almost completely disappeared,^5^ shifting therapeutic interception toward earlier stages of risk.^6^

In contrast, in ACPA-negative disease, classification and treatment decisions still largely rely on a fully expressed clinical phenotype with substantial inflammatory burden,^7 8^ as a preclinical or prodromal phase is less consistently recognisable.^2^ Nevertheless, some patients with seronegative RA may present with limited synovitis at onset^9^ or evolve from an initial UA phenotype.^10–16^ Post hoc analyses of the TREAT Early Arthralgia to Reverse or Limit Impending Exacerbation to Rheumatoid arthritis trial have even shown that approximately 30% of ACPA-negative individuals with clinically suspect arthralgia and subclinical inflammation on imaging may progress to RA.^17^

Although early intervention can improve the long-term course of ACPA-negative RA,^18 19^ and very early MTX treatment may even induce disease reversal in seronegative patients,^17 20^ balancing treatment benefits against potential risks remains challenging.^4 5 21 22^ In the absence of RA-associated autoantibodies, predicting individual outcomes remains difficult,^23^ and most individuals presenting with seronegative inflammatory arthralgia or mild synovitis will not progress to persistent RA.^4 17^

Within this framework, UA provides a pragmatic setting for investigating risk stratification. Under current classification criteria, UA has become predominantly ACPA-negative, milder and frequently self-limiting.^4^ Nevertheless, 10%–30% of patients still progress to RA or chronic arthritis.^10–16^ Classic prognostic models perform poorly in this population,^10 11^ and predictors of progression remain elusive. As a consequence of this prognostic uncertainty, management in clinical practice remains empirical and heterogeneous, often resulting in increased use of disease-modifying antirheumatic drugs (DMARDs) without clear long-term benefit.^13 24^

To address this challenge, we investigated a well-characterised cohort of contemporary ACPA-negative patients with UA managed according to a standardised treat-to-target protocol, in which treatment escalation was driven by persistent disease activity. We assessed the frequency of RA development and treatment intensification and explored whether baseline phenotype could distinguish clinically meaningful disease trajectories.

## Patients and methods

### Study population

The study population was selected from the Pavia Early Arthritis Clinic (EAC), an inception cohort of patients with new-onset inflammatory arthritis (symptoms <12 months) from the Pavia district (Italy),^25 26^ covering a population of ~550 000 inhabitants. Referral criteria have remained stable and include ≥3 swollen joints or, in case of <3 joints, a positive squeeze test or morning stiffness >30 min.^9 27^

The analysis of data was restricted to consecutive patients who satisfied the following inclusion and exclusion criteria:

- Clinical evidence of arthritis in at least one joint without fulfilment of either the 1987^28^ or the 2010^2^ RA classification criteria and absence of alternative clinical diagnoses according to the treating rheumatologist at baseline or within the first 6 months of follow-up.
- Enrolment between 2011 and 2019. The post-2010 time period was chosen to avoid potential misclassification arising from retrospective application of the 2010 RA criteria.
- Absence of ACPA based on centralised laboratory measurement on baseline sera.

### Baseline assessments

Baseline investigation followed the recommendations of the 3E (Evidence, Expertise, Exchange) initiative.^29^ Systematic history and physical examination were performed to collect information on functional status, duration of symptoms, early morning stiffness, number and pattern of tender and swollen joints. Number of swollen joints was categorised into monoarthritis, oligoarthritis (two to five joints) and polyarthritis (≥6 joints) for consistency with the derivation process of the 2010 RA classification criteria.^30^ Symmetry of swollen joints was defined as symmetric involvement of the same joint areas. The distinction between large and small joints was made in accordance with the 2010 RA classification criteria.^2^ All patients underwent conventional X-rays of the hands and feet and centralised laboratory measurement of acute phase reactants and autoantibodies. ACPA and rheumatoid factor (RF) were determined on baseline, treatment-naïve sera with a second-generation ELiA (Phadia, Uppsala, Sweden) and by nephelometry, with cut-off values of 10 U/mL for ACPA and 20 U/mL for RF, respectively. Antinuclear antibodies (ANAs) were determined by human epithelial type 2 indirect immunofluorescence with International Consensus on ANA Patterns coding.

### Treatment protocol

Patients were seen every 2 months during the first 6 months and every 3 months thereafter. The 28-joint disease activity score (DAS28) was recorded at each visit. Initial treatment consisted of hydroxychloroquine (HCQ) 200 mg twice daily for 2 months and 200 mg/day thereafter. Bridging therapy with low-dose oral prednisone (5 mg/day) for a maximum of 6 months or intra-articular injections of triamcinolone acetonide in cases of monarthritis could be prescribed according to the judgement of the treating rheumatologist.^9 25^ From month 2, patients not achieving a target of low disease activity (DAS28 <3.2) with HCQ were switched to progressively increasing doses of MTX, starting from 15 mg/week to a maximum of 25 mg/week. Sulfasalazine 2 g/day or leflunomide 20 mg/day were used in case of intolerance or contraindications to MTX. Further treatment steps included combination conventional synthetic DMARDs (csDMARDs) or initiation of a biologic or targeted synthetic DMARD, taking into account poor prognostic factors.^2^ ANA positivity was not considered a criterion for treatment initiation or treatment intensification in the absence of clinical features suggestive of a connective tissue disease.

### Outcomes

Two outcomes were assessed within 24 months of follow-up:

- RA development, defined as fulfilment of the 1987 and/or 2010 RA criteria.
- Clinically relevant disease progression, evaluated as the composite of RA development and treatment intensification. This latter was defined as escalation from HCQ to MTX or to another csDMARD because of persistent disease activity. Drug changes due to intolerance or adverse events were not counted as treatment intensification.

To reduce circularity related to the DAS28-driven treatment protocol, treatment intensification was considered clinically relevant only when active disease was accompanied by evidence of at least one swollen joint. Patients with ≥6 months of follow-up who had not developed RA or required treatment intensification by their last available visit were classified as non-events.

### Statistical analyses

Continuous variables were summarised as mean (SD) or median (IQR), and categorical variables as counts and percentages. Baseline characteristics were compared across joint-pattern groups using t-tests or analysis of variance, Mann-Whitney U or Kruskal-Wallis tests and χ^2^ or Fisher’s exact tests.

Before outcome modelling, patients were classified into three anatomical patterns: exclusive hand-joint involvement, combined hand and large-joint involvement or exclusive large-joint involvement. This classification reflected baseline joint distribution, the joint domains of the 2010 ACR/EULAR criteria^2^ and previous work on contemporary UA heterogeneity.^15^ Large and small joints were defined according to the 2010 ACR/EULAR criteria.^2^ C reactive protein (CRP) was dichotomised at >0.5 mg/dL to identify values above the normal range; a threshold of >1.0 mg/dL was additionally explored in sensitivity analysis.

Univariable logistic regression assessed associations between baseline variables and outcomes. Multivariable models included clinically selected predictors, prioritising anatomical phenotype and CRP while avoiding collinearity with component joint variables. Results are reported as ORs or adjusted ORs with 95% CIs. Discrimination was assessed by the area under the receiver operating characteristic curve (AUC). Calibration was assessed using the Hosmer-Lemeshow test; additional analyses evaluated interactions between joint phenotype and CRP >0.5 mg/dL.

Classification tree analysis used recursive partitioning to explore the hierarchical structure of predictors of the composite outcome. Candidate variables were restricted a priori to disease-related baseline features, including joint distribution, CRP, monoarticular/oligoarticular/polyarticular pattern and symmetry, as the analysis was intended to explore clinically interpretable inflammatory phenotypes rather than to develop a comprehensive prediction model. At each branch, clinically relevant candidate variables were screened, and splits were selected according to the observed separation in outcome rates and clinical interpretability.

Missing baseline data were not imputed. To assess the potential impact of loss to follow-up on outcome estimates, best-case and worst-case sensitivity analyses were performed. Analyses were performed in R. Logistic regression models used the stats package, and classification trees recursive partitioning. Two-sided p values <0.05 were considered statistically significant.

## Results

### Baseline characteristics of the study population

Of a total population of 1584 patients enrolled in the EAC between 2011 and 2019, 431 (27.2%) were initially classified as having UA at referral. During the first 6 months of follow-up, 13 were reclassified as psoriatic arthritis (PsA) (n=6), peripheral spondyloarthritis (n=3), crystal arthritis (n=2), connective tissue disease (n=1) or inflammatory osteoarthritis (n=1), and were excluded. This left 418 patients (26.4%) with contemporary UA, defined as arthritis not fulfilling either the 1987 or the 2010 RA criteria and without any alternative clinical diagnosis. Of these, 408 had complete baseline data for analysis. Six patients (1.5%) were ACPA-positive based on centralised determination at referral and were excluded (online supplemental figure 1).

Baseline characteristics of these ACPA-negative contemporary subjects with UA are shown in table 1. Patients were predominantly female (74%), with a mean (SD) age of 53 (15) years; 23.9% were older than 65 years. Symptom duration was relatively short (median (IQR) 22^12–36^ weeks), although <25% of patients were referred within 12 weeks of onset. Disease activity was mild, with a mean (SD) of SJC44 of 2.9 (2.4), and slightly increased CRP levels (mean (SD) 0.7 (1.2) mg/dL). Acute phase reactants were within the normal range in 54.5% of patients. Despite this low inflammation, disease impact was significant, with visual analogue scale (VAS) pain levels >40 mm in 67.4% of cases and Health Assessment Questionnaire (HAQ) >0.5 in 50%.

**Table 1:** Demographic and clinical characteristics of patients with ACPA-negative undifferentiated arthritis

	N=402
Age	52.9 (14.9)
Female gender	299 (74.4)
Psoriasis (family history)	31 (7.7)
Symptoms duration, weeks	22.4 (12–36.1)
Symptoms duration (weeks)	
<6	25 (6.2)
<12	91 (22.6)
Morning stiffness (min)	
<30	91 (22.6)
30–60	180 (44.8)
>60	131 (32.6)
SJC28	2.5 (1.9)
SJC44	2.9 (2.4)
Monoarthritis (1)	122 (30.3)
Oligoarthritis (2–5)	254 (63.2)
Polyarthritis (6–28)	26 (6.5)
Symmetric arthritis	162 (40.3)
TJC28	3.7 (4.9)
TJC46	5.7 (7.6)
DAS28	3.64 (1)
VAS pain	54.2 (25.6)
PGA	51.9 (25.2)
HAQ	0.63 (0.53)
ESR	15.1 (15.1)
CRP	0.7 (1.2)
RF	18 (4.5)
ANA	186 (46.3)
SHS erosion score ≥1	30 (7.5)

ACPA, anticitrullinated protein autoantibodies; ANA, antinuclear antibody; CRP, C reactive protein; DAS28, 28-joints Disease Activity Score; ESR, erythrocyte sedimentation rate; HAQ, Health Assessment Questionnaire; PGA, Patient Global Assessment; RF, rheumatoid factor; SHS, Sharp van der Heijde Score; SJC, swollen joint count; TJC, tender joint count; VAS, visual analogue scale.

About 8% had a family history of PsA, but none met the ClASsification criteria for Psoriatic ARthritis criteria. Although ANAs were positive in ~45% of the population, the titre was low (≤1:160) in 85% of cases and no patient had signs or symptoms of a connective tissue disease. RF was found in 18 patients (4.5%), but in only two cases (0.5%) did the titre exceed three times the upper limit of normal. At least one radiographic erosion was found in 7.5% of the cases.

### Patterns of joint involvement

The involvement of different joint groups is shown in figure 1A. Collectively, the wrists were the most frequently swollen joints, followed by metacarpophalangeal and large joints. Swelling of the proximal interphalangeal joints was rarer, and metatarsophalangeal joints were mostly unaffected. The combination of involved joint groups is shown in figure 1B. The majority of the patients (75.1%) had exclusive involvement of hand joints (wrists and/or small joints) (group 1), 19.2% had combined involvement of hand and large joints (group 2) and the remaining 5.7% had exclusive involvement of large joints (group 3).

**Figure 1:**
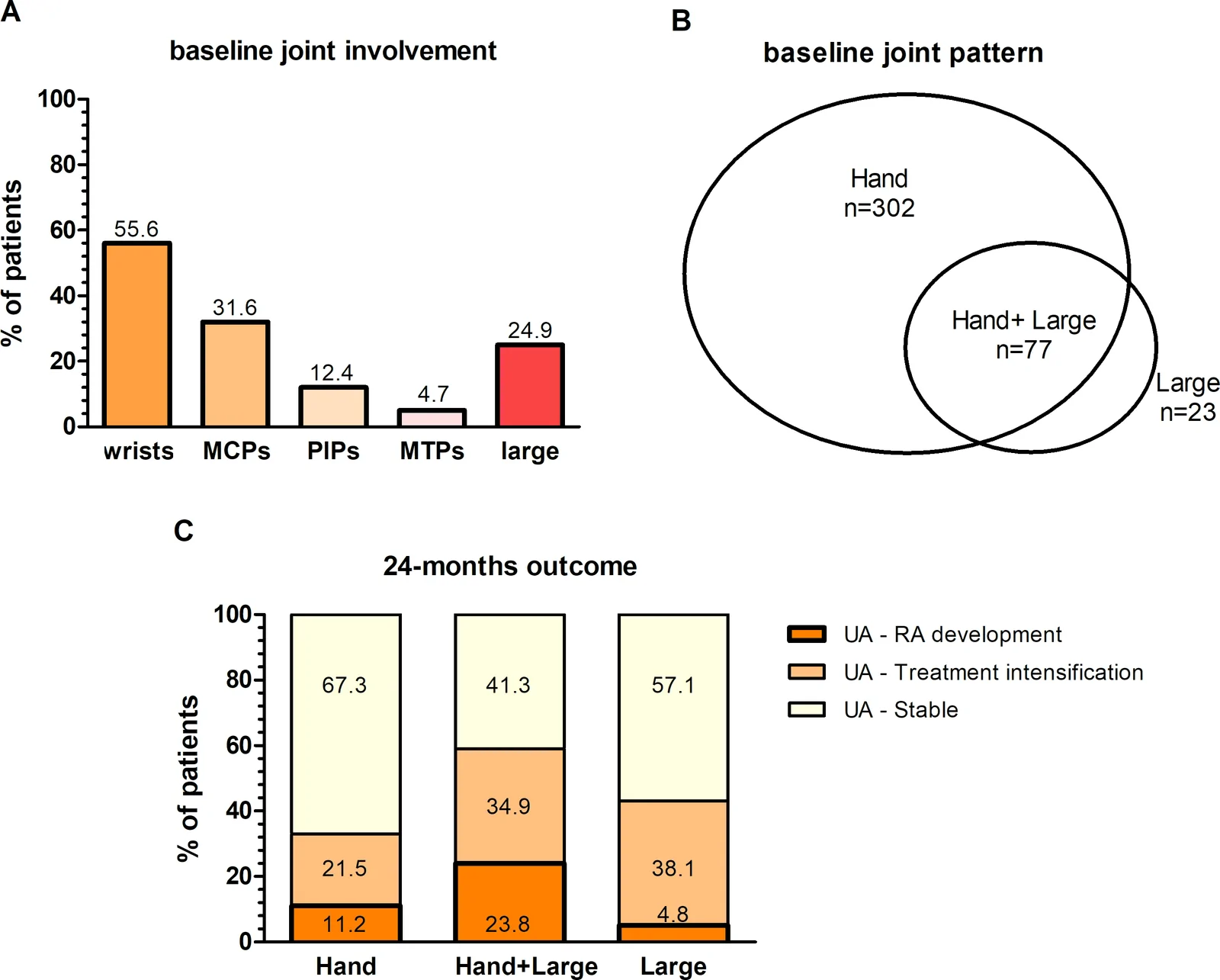
Baseline joint distribution and 24-month outcomes according to anatomical pattern. (**A**) Frequency of swollen joint involvement at baseline according to anatomical site. Wrists were the most frequently involved joints, followed by MCPs and large joints, whereas PIP and MTP involvement was uncommon. (**B**) Baseline classification of patients according to the anatomical pattern of arthritis: exclusive hand-joint involvement, combined hand and large-joint involvement and exclusive large-joint involvement. Hand involvement included wrists and/or small joints of the hands. (**C**) Distribution of 24-month outcomes across anatomical patterns among patients with available follow-up data. Outcomes were categorised as RA development, treatment intensification without fulfilment of RA criteria or stable UA. MCPs, metacarpophalangeal joints; MTPs, metatarsophalangeal joints; PIPs, proximal interphalangeal joints; RA, rheumatoid arthritis; UA, undifferentiated arthritis.

Table 2 shows baseline characteristics according to joint-pattern group. Irrespective of hand-joint involvement, patients with large-joint arthritis (groups 2 and 3) were younger and had greater functional impairment, as reflected by higher HAQ scores. Access to secondary care was faster, with approximately one-third being referred to the EAC within the first 12 weeks of symptom onset, as compared with 20% of patients with exclusive arthritis of the hands (group 1). Compared with the other groups, patients with exclusive large-joint involvement (group 3) were younger, numerically more frequently male and most often presented with monarthritis. Among patients with hand-joint involvement (groups 1 and 2), those with concomitant large-joint arthritis were younger, had shorter disease duration and greater functional impairment and more frequently presented with higher numbers of swollen joints. Symmetric involvement was comparable.

**Table 2:** Demographic and clinical characteristics of patients with ACPA-negative undifferentiated arthritis according to the pattern of joint involvement

	Group 1 hand N=302	Group 2 hand+large N=77	Group 3 large N=23
Age	54.7 (15.2)*	50.2 (15)*†	42.2 (15.3)*†
Female gender	225 (74.5)	59 (76.6)	15 (65.2)
Psoriasis (family history)	21 (7)	7 (9.1)	3 (13)
Symptoms duration, weeks	24.4 (12.3–36.6)	22.4 (11–33.1)	21.3 (9.2–28.4)
Symptoms duration (weeks)			
<6	10 (3.3)*	12 (15.6)*	3 (13.0)
<12	60 (19.9)*	24 (31.2)*	7 (30.4)
Morning stiffness (min)			
<30	71 (23.5)	14 (18.2)	6 (26.1)
30–60	130 (43)	38 (49.4)	12 (52.2)
>60	101 (33.4)	25 (32.5)	5 (21.7)
SJC28	2.4 (1.1)*	2.7 (2.2)†	1.1 (0.7)*†
SJC44	2.4 (1.6)*	3.4 (2.3)*†	1.5 (0.9)*†
Monarthritis	104 (34.4)*	0 (0)*†	18 (78.3)*†
Oligoarthritis	180 (59.6)*	69 (89.6)*†	5 (21.7)*†
Polyarthritis	18 (6)	8 (10.4)	0 (0)
Symmetric arthritis	124 (41.1)*	33 (42.9)	5 (21.7)*
TJC28	3.9 (5.5)	3.7 (4.3)	1.9 (2.5)
TJC46	5.7 (8.2)	6.2 (6.9)†	3.2 (3.8)†
DAS28	3.6 (1.1)	3.6 (1)	3.3 (0.8)
VAS pain	53.8 (25.2)	55.1 (26.3)	55.6 (22.1)
PGA	52.3 (25.6)	52.8 (26.8)	57.3 (19.6)
HAQ	0.56 (0.47)*	0.71 (0.57)*	0.69 (0.41)*
ESR	14.2 (15.4)	16.5 (18.5)	13.8 (14.5)
CRP	0.6 (1)*	0.7 (1.1)	1 (1.5)*
RF	14 (4.6)	3 (3.9)	1 (4.3)
ANA	134 (44.4)	39 (50.6)	13 (56.5)
SHS erosion score ≥1	23 (7.6)	6 (7.8)	1 (4.3)

* P<0.05 group 1 vs others.

† P<0.05 group 2 vs group 3.

ACPA, anticitrullinated protein autoantibodies; ANA, antinuclear antibodies; CRP, C reactive protein; DAS28, 28-joints Disease Activity Score; ESR, erythrocyte sedimentation rate; HAQ, Health Assessment Questionnaire; PGA, Patient Global Assessment; RF, rheumatoid factor; SHS, Sharp van der Heijde Score; SJC, swollen joint count; TJC, tender joint count; VAS, visual analogue scale.

### Outcomes

#### Development of definitive diagnoses at follow-up

Fifty-eight patients (14.4%) were lost before completing a follow-up of at least 6 months. Loss to follow-up increased to 21.9% at 12 months and 35.8% at 24 months. None of the baseline characteristics significantly influenced study discontinuation, apart from a trend towards younger age (data not shown).

During the 24-month observation period, 45 (13.1%) of the 344 patients with available outcome data fulfilled RA classification criteria. As shown in figure 1C, RA development occurred almost exclusively in patients presenting with hand arthritis (groups 1 and 2), with a significantly higher frequency in those with concomitant large-joint involvement. In sensitivity analyses using best-case and worst-case imputations for patients lost to follow-up, the excess risk of RA progression associated with combined hand and large-joint involvement remained consistent, at approximately 10–12 percentage points compared with the other joint-pattern groups.

#### Treatment intensification

All the 45 patients fulfilling RA criteria at follow-up started MTX, and 86/299 additional patients who remained classified as UA (28.8%) required escalation to csDMARDs (MTX in 87.2% of the cases) because of persistent disease activity (DAS28 >3.2). The majority of treatment intensifications (58.1%) occurred within month 6 from baseline, and were significantly more common in patients presenting with large-joint arthritis, with further risk enrichment in case of concomitant hand involvement (figure 1C). Extreme-case sensitivity analyses accounting for loss to follow-up again confirmed significantly higher risk in group 2 (p<0.001).

#### Predictors of RA development and treatment intensification

In univariable analysis, baseline demographic and clinical variables such as gender, symptom duration, morning stiffness, joint counts or symmetry were not significantly associated with RA development or treatment intensification (table 3). Instead, prognostic value resided primarily in the anatomical pattern of arthritis. Patients with combined hand and large-joint involvement had a higher risk of both RA development (OR 2.49, 95% CI 1.24 to 5.00) and treatment intensification (OR 2.93, 95% CI 1.67 to 5.15) compared with those with exclusive hand involvement. By contrast, isolated large-joint arthritis did not increase the risk of either outcome. Interestingly, large-joint swelling was more strongly linked to the composite endpoint than to RA alone. Similarly, markers of inflammatory and symptomatic burden, particularly CRP levels and VAS pain, were predominantly related to treatment intensification, regardless of RA status.

**Table 3:** Predictors of RA development and/or treatment intensification in ACPA-negative undifferentiated arthritis—univariable analysis

	RA development			RA development/Treatment intensification		
	OR	95% CI	P value	OR	95% CI	P value
Demographics						
Age	1.00	0.98 to 1.02	0.86	1.00	0.98 to 1.01	0.64
Age (years)						
≤44	ref			ref		
45–64	0.99	0.47 to 2.10	0.98	1.20	0.70 to 2.04	0.51
≥65	0.79	0.32 to 1.94	0.61	0.87	0.47 to 1.62	0.67
Female gender	1.32	0.63 to 2.79	0.46	0.94	0.58 to 1.54	0.81
Symptom history						
Symptoms duration	0.99	0.98 to 1.01	0.30	1.00	0.99 to 1.00	0.33
Symptoms duration <12 weeks	0.68	0.30 to 1.56	0.36	0.85	0.49 to 1.47	0.56
Stiffness ≥30 min	0.60	0.25 to 1.47	0.27	0.94	0.51 to 1.76	0.86
Stiffness ≥60 min	0.76	0.38 to 1.53	0.44	0.86	0.54 to 1.41	0.58
Joint phenotype						
Swollen large joint	1.84	0.92 to 3.65	0.08	2.51	1.47 to 4.30	<0.001***
Swollen hand	3.74	0.49 to 28.68	0.20	0.89	0.36 to 2.18	0.80
MTP squeeze	0.82	0.10 to 6.73	0.85	0.40	0.08 to 1.92	0.25
Hand	ref			ref		
Large	0.40	0.05 to 3.08	0.38	1.54	0.63 to 3.81	0.35
Hand+large	2.49	1.24 to 5.00	0.01*	2.93	1.67 to 5.15	<0.001***
Monarthritis	ref			ref		
Oligoarthritis	0.92	0.46 to 1.85	0.81	0.92	0.57 to 1.49	0.74
Polyarthritis	1.77	0.57 to 5.49	0.33	1.63	0.67 to 3.98	0.28
Symmetric involvement	1.20	0.62 to 2.33	0.59	1.41	0.90 to 2.22	0.14
Joint counts						
SJC28	1.12	0.97 to 1.30	0.11	1.04	0.93 to 1.16	0.54
SJC44	1.05	0.93 to 1.19	0.43	1.02	0.93 to 1.11	0.74
TJC28	1.00	0.94 to 1.07	0.97	1.00	0.96 to 1.04	0.99
TJC46	1.00	0.96 to 1.04	0.90	1.01	0.98 to 1.03	0.70
Patient-reported						
VAS pain	1.02	0.99 to 1.03	0.06	1.01	1.00 to 1.02	0.02*
PGA	1.01	0.99 to 1.02	0.50	1.01	0.99 to 1.02	0.37
HAQ	1.29	0.63 to 2.64	0.49	0.92	0.54 to 1.56	0.75
Laboratory and imaging						
ESR	1.00	0.98 to 1.02	0.84	1.00	0.99 to 1.02	0.56
ESR >15	0.98	0.50 to 1.88	0.92	1.17	0.74 to 1.84	0.50
CRP	1.03	0.77 to 1.39	0.82	1.26	1.01 to 1.58	0.04*
CRP >0.5	1.68	0.89 to 3.19	0.11	1.89	1.20 to 2.98	0.006**
RF	1.11	0.24 to 5.14	0.89	0.64	0.20 to 2.08	0.46
ANA	1.01	0.52 to 1.98	0.98	1.09	0.68 to 1.74	0.72
SHS erosion score ≥1	1.06	0.35 to 3.19	0.92	0.99	0.45 to 2.17	0.98

*p<0.5 **p<0.01 ***p<0.001

ACPA, anticitrullinated protein autoantibodies; ANA, antinuclear antibodies; CRP, C reactive protein; ESR, erythrocyte sedimentation rate; HAQ, Health Assessment Questionnaire; MTP, metatarsophalangeal joints; PGA, Patient Global Assessment; RA, rheumatoid arthritis; ref, reference; RF, rheumatoid factor; SHS, Sharp van der Heijde Score; SJC, swollen joint count; TJC, tender joint count; VAS, visual analogue scale.

Multivariable analysis confirmed that the baseline joint phenotype was an independent predictor of outcomes (table 4). In the final model, combined hand and large-joint arthritis significantly predicted subsequent RA development compared with exclusive hand involvement (adjusted OR 2.68, 95% CI 1.29 to 5.57), while isolated large-joint involvement did not. CRP >0.5 mg/dL showed a non-significant positive association. The discriminative performance of the model was modest, with an AUC of 0.681. For the composite outcome of RA development or treatment intensification, the combined anatomical phenotype demonstrated an even stronger independent association (adjusted OR 3.32, 95% CI 1.77 to 6.24), and increased CRP and VAS pain also retained independent significance. Model discrimination was higher, with an AUC of 0.695. Calibration showed no evidence of substantial lack of fit. The association with both outcomes remained significant after additional adjustment for the number of swollen joints or polyarticular involvement. Adding other demographic or clinical baseline variables, such as age, gender, symptom duration and HAQ, only minimally changed discrimination, while no significant interaction between joint phenotype and CRP was detected. A different threshold of CRP >1.0 mg/dL was relatively infrequent (17% of patients), and its association with the composite outcome remained directionally positive but did not reach statistical significance (OR 1.53, 95% CI 0.87 to 2.72). Sensitivity analyses including patients lost to follow-up supported the consistency of the main findings. Combined hand and large-joint involvement maintained a directional association in the best-case scenario and a significant independent association in the worst-case scenario, while elevated CRP remained independently associated under both assumptions.

**Table 4:** Predictors of RA development and/or treatment intensification in ACPA-negative undifferentiated arthritis—multivariable analysis

	RA development			RA development/Treatment intensification		
	adjOR	95% CI	P value	adjOR	95% CI	P value
Large-joint only versus hand-joint only	0.30	0.04 to 2.36	0.25	1.23	0.49 to 3.14	0.66
Hand+large-joint versus hand-joint only	2.68	1.29 to 5.57	0.008**	3.32	1.77 to 6.24	<0.001***
CRP >0.5 mg/dL	2.18	0.97 to 4.87	0.06	2.43	1.26 to 4.69	0.008**
VAS pain, per mm	1.02	1.00 to 1.04	0.07	1.02	1.00 to 1.03	0.008**

**p<0.01 ***p<0.001

ACPA, anticitrullinated protein autoantibodies; adjOR, adjusted OR; CRP, C reactive protein; RA, rheumatoid arthritis; VAS, visual analogue scale.

### Classification tree analysis

Classification tree analysis was used to explore the sequential structure of baseline predictors for the composite outcome of RA development or treatment intensification (figure 2). In keeping with the multivariable findings, the first split was based on the presence of large-joint involvement. The outcome occurred in 54.8% of patients with large-joint involvement compared with 32.7% of patients without large-joint involvement. Among patients with large-joint involvement, hand arthritis further enriched for outcome occurrence, with event rates increasing from 42.9% to 58.7%. Conversely, among patients without large-joint involvement, inflammatory burden became the most informative subsequent discriminator: CRP >0.5 mg/dL identified a higher-risk subgroup (41.1% vs 28.2%). Finally, within the low-CRP/no-large-joint branch, swollen wrist involvement identified a numerically higher event rate (36.6% vs 20.5%), although this terminal split was less robust than the preceding nodes and only exploratory. Monoarticular/oligoarticular/polyarticular pattern and symmetry of swollen joints did not provide stable additional discrimination. Following the tree into its terminal branches showed that large-joint involvement alone accounted for approximately one-third of all outcome events, while the no-large-joint/CRP >0.5 mg/dL branch captured an additional quarter of events. When these two main high-risk branches were considered together, the model identified 64% of outcome events. The corresponding positive predictive value (PPV) was 48%, while negative predictive value (NPV) was 72%. Apparent classification accuracy was modest, around 60%, supporting the use of the tree as a clinically interpretable stratification tool rather than as a stand-alone predictive classifier.

**Figure 2:**
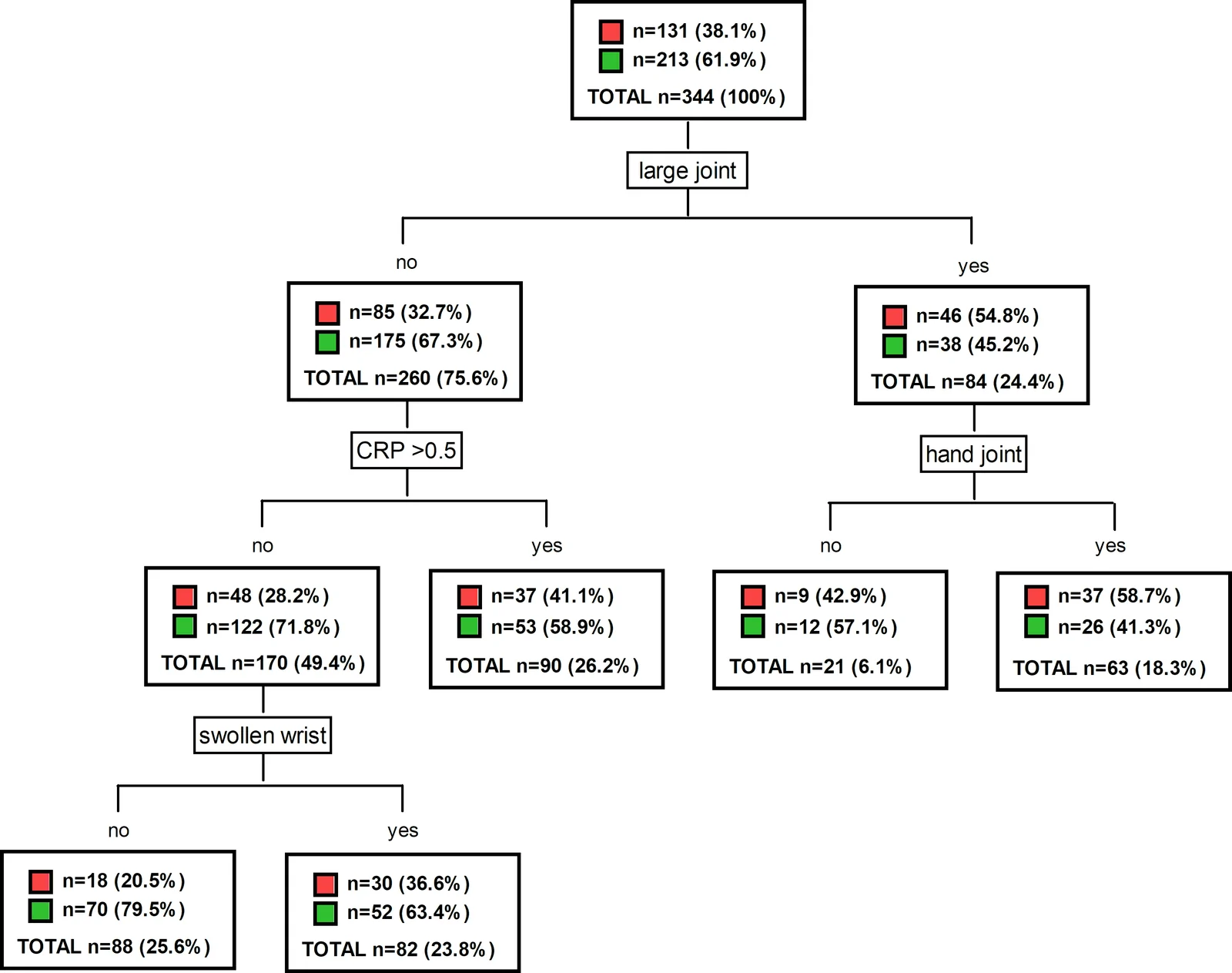
Classification tree for RA development or treatment intensification in ACPA-negative undifferentiated arthritis. Classification tree analysis was used to explore the hierarchical structure of baseline predictors of the composite outcome of RA development or treatment intensification among patients with available follow-up data. The first split was based on the presence of large-joint involvement. Among patients with large-joint arthritis, concomitant hand involvement further enriched the probability of outcome occurrence. Among patients without large-joint involvement, CRP elevation identified a higher-risk subgroup. The terminal split based on swollen wrist involvement should be considered exploratory and less robust than the preceding branches. Red boxes indicate patients who developed RA or required treatment intensification; green boxes indicate patients who remained stable. Percentages within each node refer to the proportion of patients with or without the composite outcome within that node, while total percentages refer to the proportion of the whole analysed population. ACPA, anticitrullinated protein antibodies; CRP, C reactive protein; RA, rheumatoid arthritis.

## Discussion

Within this cohort of contemporary ACPA-negative UA managed under a standardised treat-to-target protocol, 13% of patients developed criteria-defined RA over 24 months, while 38% reached the composite endpoint of RA evolution or treatment intensification for persistent disease activity. Disease progression was not captured by conventional variables traditionally embedded in validated prediction rules. Instead, prognosis was primarily determined by the anatomical topography of synovitis: combined hand and large-joint involvement independently predicted both RA development and treatment intensification, while CRP refined the risk of therapeutic escalation. Classification tree analysis generated an intuitive hierarchical model, with large-joint involvement as the primary discriminator and subsequent stratification by concomitant hand involvement or CRP elevation.

The baseline composition of our cohort provides a specific setting to investigate UA. Unlike historical studies applying the 2010 ACR/EULAR criteria retrospectively,^10–12 14^ our post-2010 enrolment ensured a truly contemporary and representative population. Furthermore, because classic RA predictors such as autoantibodies are inherently unhelpful in contemporary UA,^10 11 31^ focusing on ACPA-negative individuals addressed the fundamental challenge of stratifying those who remain at risk of progression.

Despite differences in case definitions, recruitment periods and referral pathways, the baseline phenotype of our cohort closely resembled other 2010-UA populations, which generally included a proportion of ACPA-positive patients.^10–13 16^ We could confirm the low-grade inflammatory profile of seronegative UA, with few swollen joints and normal acute-phase reactants in most patients. This mild inflammatory burden contrasted with substantial patient-reported impact. Although functional disability remains underinvestigated in this setting, our findings are consistent with the Treatment in the Rotterdam Early Arthritis CoHort trial, which demonstrated that patient-reported outcomes in UA closely paralleled those of autoantibody-positive RA.^32^ This corroborates that pain and functional impairment can decouple from objective inflammation^33^ even in early seronegative undifferentiated disease. Whether functional disability might represent an additional outcome in contemporary UA alongside the traditional focus on RA progression warrants consideration.

The distribution of synovitis highlighted the topographical heterogeneity of contemporary UA. Arthritis predominantly involved the hands,^10–12 15^ whereas the feet were rarely affected. Dakkak *et al*^34^ similarly reported clinical and MRI foot abnormalities in only a minority of patients with 2010-UA. This pattern also parallels seronegative RA, in which forefoot inflammation on MRI is less extensive,^35^ and radiographic erosions accumulate less prominently than in seropositive disease.^36^ Limited foot involvement may therefore represent a shared anatomical feature of autoantibody-negative inflammatory arthritis. Recent data in early RA further showed that a foot-predominant phenotype was relatively enriched for ACPA positivity, although joint topography retained prognostic relevance beyond autoantibody status.^37^ The rarity of foot involvement in our cohort precluded a sufficiently powered analysis of hand-dominant versus foot-dominant phenotypes, which should be explored in larger cohorts of contemporary ACPA-negative UA. Isolated large-joint arthritis was also uncommon, contrasting with higher frequencies reported elsewhere,^15 38^ possibly because our diagnostic filtering excluded patients who developed alternative diagnoses during early follow-up, and our referral criteria give more weight to oligo-polyarticular presentation. Nevertheless, large-joint swelling affected approximately one-quarter of patients and usually coexisted with hand involvement. We therefore assessed large-joint disease within its anatomical context. Building on den Hollander *et al* latent class analysis, which separated contemporary UA phenotypes mainly by joint count and distribution,^15^ we defined three pragmatic phenotypes: exclusive hand involvement, combined hand/large-joint involvement and isolated large-joint disease. This framework retains the main dimensions of previous models while remaining directly feasible in routine baseline assessment.

The 13% rate of RA development in our cohort was at the lower end of contemporary 2010-UA studies,^10 11 14 16^ likely because ACPA-positive patients were excluded, and closely matched the 14% reported in autoantibody-negative UA.^15^ Because RA classification captures only part of disease persistence, we also evaluated escalation to a more intensive DMARD strategy. Unlike studies in which DMARD initiation reflected expert opinion or routine prescribing,^13^ escalation in our cohort followed predefined treat-to-target rules. Even using this broader endpoint, treatment intensification occurred in 38%, only slightly less than in mixed 2010-UA cohorts.^13^ Thus, although ACPA-negative UA is not simply preclassifiable RA, a substantial subset still develops persistent disease under standardised follow-up.

After exclusion of ACPA-positive patients, classical predictors in contemporary UA remain notably limited.^10–12 16 31 38^ Earlier tools, such as the Leiden rule,^39^ were developed in pre-2010-UA populations and incorporated demographic, clinical and laboratory variables, including autoantibodies, with negligible predictive performance in contemporary UA.^10 11^ Polyarthritis remains informative for identifying patients requiring DMARD intensification,^14 15^ but its low prevalence limits its ability to capture most persistent cases.^12 14 15^ Advanced imaging can improve prediction by detecting subclinical synovitis or tenosynovitis,^14 34 40 41^ but limited access, particularly to MRI, restricts systematic use. Our findings indicate that combined large-joint and hand-joint synovitis is more prognostically informative than absolute joint count. Large-joint swelling was associated with greater baseline disease burden and increased risk of RA development or treatment intensification, with a PPV of 54.8% and an NPV of 67.3% for the composite outcome. This is consistent with early RA literature linking large-joint disease to poorer prognosis.^42^ More importantly, our findings translate previous imaging observations into routine practice. While MRI-detected small-joint synovitis independently predicted progression in large-joint UA,^41^ our data show that concomitant, clinically detectable hand involvement captures this same prognostic concept, acting as a reliable, visible marker of more systemic inflammation. Among patients without large-joint involvement, CRP provided an additional level of stratification. Although these patients had a lower overall risk, CRP elevation increased the probability of treatment intensification from 28% to 41%. Again, this finding parallels MRI data in contemporary UA, where subclinical tenosynovitis improved prediction beyond clinical joint counts.^14^ Notably, in that setting MRI negativity had a high NPV for RA development, similarly to CRP in our study.

Strengths of this study include a well-characterised contemporary cohort, centralised ACPA testing, predefined treatment escalation rules and the high real-world applicability of an approach based entirely on routine clinical and laboratory metrics. Some limitations warrant consideration. Our findings require external validation. Referral criteria may have selected for more symptomatic disease, potentially limiting generalisability to very mild UA managed in primary care. Loss to follow-up, although comparable with previous cohorts,^12^ may have introduced attrition bias towards higher disease severity if patients in remission were more likely to disengage; however, sensitivity analyses helped mitigate the potential impact of this bias. The lack of systematic imaging precluded evaluating subclinical inflammation across clinical phenotypes. While using DMARD escalation as an endpoint aligns with the methodology of RA criteria development,^2^ it may have resulted in diagnostic misclassification of some non-RA cases. Although treatment intensification required persistent disease activity, subjective DAS28 components may still have influenced treatment decisions, potentially introducing some misclassification. Finally, given its moderate model discrimination, our proposed stratification should be considered a pragmatic clinical framework rather than a definitive prognostic tool.

In conclusion, contemporary ACPA-negative UA remains a heterogeneous condition, but its prognosis is not random. In a standardised treat-to-target setting, clinically relevant progression is structured by the anatomical distribution of synovitis, with combined hand and large-joint involvement identifying a high-risk phenotype and CRP refining risk among patients without large-joint arthritis. Although the predictive ability of these variables remains modest, these findings support a pragmatic, low-cost approach to risk stratification and may help balance overtreatment and undertreatment in a population for which conventional predictors are poorly informative.

## Supplementary material

10.1136/rmdopen-2026-007443 online supplemental file 1

## Data Availability

Data are available on reasonable request.
